# The Content of Secondary Metabolites and Antioxidant Activity of Wild Strawberry Fruit (*Fragaria vesca* L.)

**DOI:** 10.1155/2015/831238

**Published:** 2015-10-11

**Authors:** Magdalena Dyduch-Siemińska, Agnieszka Najda, Jan Dyduch, Magdalena Gantner, Kamila Klimek

**Affiliations:** ^1^Department of Genetics and Horticultural Plant Breeding, University of Life Sciences in Lublin, Akademicka Street 15, 20-068 Lublin, Poland; ^2^Department of Vegetable and Medicinal Plants, University of Life Sciences in Lublin, K. Leszczyńskiego Street 58, 20-068 Lublin, Poland; ^3^Division of Engineering in Nutrition, Warsaw University of Life Sciences, Nowoursynowska Street 159, 02-776 Warsaw, Poland; ^4^Department of Applied Mathematics and Informatics, University of Life Sciences in Lublin, Głęboka Street 28, 20-612 Lublin, Poland

## Abstract

Chemical analyses carried out in 2011–2013 aimed at evaluating the contents of flavonoids, free phenolic acids, tannins, anthocyanins, and antioxidant activity (%) by means of DPPH radical neutralization ability in fresh and air-dried fruits of three wild strawberry cultivars. Examinations revealed differences in contents of biologically active substances determined in raw versus dried material depending on the cultivar. Mean concentrations of flavonoids and tannins were highest in raw fruits of “Baron von Solemacher” cv., which amounted to 1.244 mg·g^−1^ and 6.09%, respectively. Fresh fruits of “Regina” cv. were characterized by the highest average content of phenolic acids and anthocyanins: 4.987 mg·g^−1^ and 0.636 mg·100 g^−1^. The pattern of mean contents of biologically active substances analyzed in air-dried fruits was similar. Significant differences in abilities to neutralize the DPPH radical to diphenylpicrylhydrazine by extracts made of examined wild strawberry fruits were also indicated.

## 1. Introduction

*Fragaria vesca* L. is a well-known and valuable plant species; however, its cultivation is still much poorly spread [1]. Currently grown cultivars of* Fragaria vesca* are derived from a wild species, which can be found in woods and grasslands in Europe, Western Asia, North America, and temperate areas in Chile [2].* Fragaria vesca *ssp.* vesca* have been cultivated for centuries in European gardens. Their widespread temperate growing range, self-compatibility, and long history of cultivation, coupled with selection for favorable recessive traits such as day neutrality, nonrunnering, and yellow-fruited forms, offer extensive genotypic diversity [3]. Despite high price, wild strawberries fruits are a product that is highly appreciated by consumers [1]. Their natural aspect, color, nutritional values, and high natural antioxidant compounds content are their most attractive characteristics. A high percentage of these fruits are sold as a frozen product, which is used in the manufacturing of cakes, ice creams, or milk desserts [1, 4]. There are an increasing demand for fresh berries and, consequently, a need to increase their distribution ratio and shelf life [1]. *Fragaria* spp. is unusual in what is called the fruit actually originates from the expansion of the flower base (the receptacle) as a pseudocarp, with the real one seeded fruits (achenes) on the epidermal layer [5–7]. Wild strawberry fruits ripen during 3-4 weeks after flowers develop, although the period greatly depends on the weather conditions. Many epidemiological studies have shown that a higher consumption of fruit and vegetables is associated with the prevention of chronic diseases such as diabetes, heart disease, and certain cancers. Apart from essential nutrients, fruit and vegetables also contain a variety of different phytochemicals that can act as antioxidants, prevent oxidation, and also exhibit other bioactive physiological properties. Different antioxidants, such as flavonoids, phenolic acids, carotenoids, and ascorbic acid, have been proposed to act anticarcinogenically [8–13]. Red, spherical, sweet taste fruits (*Fragariae fructus*) with unforgettable flavor are one of the raw materials achieved from wild strawberry [13]. According to much of the research done, wild strawberry fruits have high antioxidant activity, which has been linked to their content of phenolic compounds [4, 14].

Small fruit breeding programs are currently used to acquire new cultivars improved for specific agronomic (yield and size), qualitative (firmness, sugars content, and acidity), and sensorial (colour and aroma) characteristics, all combined to increased disease resistance and plant adaptability. Nowadays, besides all these parameters, it is necessary to look for the specific bioactive components well known for their effect on human health. This aspect is now highly requested by the consumer [15]. Flavonoids and phenolic acids are the most common phenolic compounds in small fruits with strong antioxidant capacity [13, 16–18]. Taking this into account, the following study was carried out to make chemical analyses aim at evaluating the contents of flavonoids, free phenolic acids, tannins, anthocyanins, and antioxidant activity (%) by means of DPPH radical neutralization ability in fresh and air-dried fruits of three wild strawberry cultivars. The aim of these studies was to identify cultivars which were characterized by the highest content of investigated biologically active substances and the highest ability to free radicals elimination. Fruits of these cultivars will be characterized by the highest prohealth properties. The results from the performed experiments will also have practical application during the breeding program preparation in order to obtain new cultivars of this species.

## 2. Experiment

The study material consisted of fruits collected from three cultivated wild strawberry cultivars: “Baron von Solemacher,” “Yellow Wonder,” and “Regina” originating from agrotechnical experiments carried out at Department of Vegetable and Medicinal Plants, University of Life Sciences, Lublin (Poland, 51°23′ N, 22°56′ E). Seeds of tested cultivar were sown manually on March 5th, 2010, into boxes filled with a substrate (peat substrate) and covered with thin sand layer. After emergence and forming 2-3 true leaves, the seedlings were transferred into boxes at 5 × 3.5 cm spacing. Plants were set into their permanent place on June 20th, 2010, in plots of 7.5 m^2^ area (2.0 × 3.75 m) at 40 × 25 cm spacing (14 plants per row, i.e., 9.3 plants·m^−2^) in three replicates. The agrotechnical experiment was carried out on dusty soil characterized by good abundance in nutrients and neutral reaction. Soil under wild strawberry cultivation was prepared according to commonly accepted recommendations applying manure (40 kg·ha) for the forecrop (onion). Phosphorus, potassium, and magnesium fertilizers were used before seedling planting at the following amounts: 80 kg·ha—P_2_O_5_; 100 kg·ha—K_2_O. Starter rate (N—30 kg·ha) was applied, when the seedling was taken the roots. The wild strawberry plantation was regularly manually deweeded. First harvest was in second growing years. The fruit harvest was carried out at the full of fruiting stage in years 2011–2013. Fruits were harvested once in the early morning, in June every year.

Weather conditions during growth and studies upon* Fragaria vesca* are presented in Table 1.

Directly after the harvest, part of material was subject to laboratory analyses as raw, while another part was dried out. The drying process was performed in a drying facility at 40°C till the moment of a constant air-dried fruit weight achievement. Raw material was subject to determinations of dry matter (%) by means of drier method [19], weight loss after drying-moisture (%) [20] content flavonoids (mg·g^−1^) [20], sum of phenolic acids (mg·g^−1^) [21], tannins (%) [20], anthocyanins (mg·100 g^−1^) [22], and antioxidant capacity (%) as an ability to neutralize the DPPH radicals [23]. Biochemical analysis was performed in Laboratory for Vegetable and Herbal Material Quality at the Department of Vegetable and Medicinal Plants, University of Life Sciences, Lublin.

### 2.1. Dry Matter

Dry matter determination was carried out by means of drier method according to Charłampowicz [19]. Aliquots of about 1 g (0.0001 g accuracy) of raw and ground fruits were weighed. Samples were placed in a drier and dried at 105°C for 6 hours. The drying process was repeated till the constant weight of samples (difference between two subsequent weighings should not be greater than 0.5 mg). The difference of weights before and after drying was the water loss, and then the result was recalculated onto the percentage of dry matter. Determinations were made in three replicates.

### 2.2. Weight Loss after Drying-Moisture Content

The loss after drying was determined by means of gravimetric method according to Polish Pharmacopoeia VII [20]. Samples of 1 g of three cultivars of ground wild strawberry fruits were weighed in vessels. Samples were then placed in a drier at 105°C and dried for 2 hours; after that they were cooled to ambient temperature in desiccator over silica gel and weighed again. The drying was repeated until the constant weight (difference between two subsequent weighings should not be greater than 0.5 mg). The difference of weights before and after drying was the water loss (moisture content); all determinations were made in 3 replicates.

### 2.3. Total Flavonoids Estimation

Total flavonoids were estimated according to the spectrophotometric method of Christ and Müller [20] after their extraction, as recommended by the European Pharmacopoeia. For this purpose, 2.0 g of crushed fruit was added to a round-bottomed flask; 20 mL of acetone, 2 mL of HCl (281 g·L^−1^), and 1 mL of methenamine (5 g·L^−1^) were then added and the mixture was maintained for 30 min under reflux on a water bath. The hydrolysate was filtered through cotton wool into a volumetric flask of 100 mL, then placed in a flask together with the cotton pellet and 20 mL of acetone, and refluxed for 10 min. Next, 20 mL of solution was dispensed into a separatory funnel with 20 mL of water and extracted with ethyl acetate in 15 mL portions 3 times with 10 mL. The combined organic layers were washed twice with 40 mL of water, filtered into a volumetric flask of 50 mL, and supplemented with ethyl acetate. To determine flavonoid content, 2 samples were prepared: to 10 mL of the stock solution 2 mL of a solution of aluminum chloride (20 g·L^−1^) was added, supplemented with a mixture (1 : 19) of acetic acid (1.02 kg·L^−1^) and methanol (25 mL). To prepare the comparative solution, stock was supplemented with 10 mL of a mixture (1 : 19) of acetic acid (1.02 kg·L^−1^) and methanol (25 mL). After 45 min, the absorbance of the solutions was read at *λ* = 425 nm on HITACHI U-2900 spectrophotometer using the reference solution for comparison. Samples were analyzed in 3 replicates. The total content of flavonoids (mg·g^−1^) was expressed as quercetin QE equivalent according to the following formula:

(1) $$\begin{array}{c} X=\frac{k\times A}{m}, \end{array}$$

where *X* are total flavonoids (mg·g^−1^); *A* is the absorbance of the solution being studied; *k* is the convection factor for quercetin and equal to 8.750; *m* is the sample with the raw material (g) which was the amount of fresh and dry material.

### 2.4. Total Phenolic Acids Estimation

Total phenolic acids estimation was carried out according to Arnov method [21], which corresponds to the recommendations of the European Pharmacopoeia. To 5.0 g of homogenized raw material placed in a round-bottomed flask 20 mL of methanol was added and the mixture was heated for 30 min at 70°C in a water bath at reflux. The hydrolysate was filtered through a hard filter paper into an Erlenmeyer flask of 100 mL. The filtered medium was returned to the round-bottomed flask with 20 mL of methanol and heated at reflux for 30 min. This process was repeated 3 times. The combined filtrates were taken to the tube with 1 mL of blueberry extract, 1 mL of 0.5 N hydrochloric acid, 1 mL of Arnov reagent, and 1 mL of 1 N sodium hydroxide, made up to 10 mL with distilled water. The absorbance was measured at *λ* = 490 nm. The total phenolic acid content, expressed as acid equivalent weight of caffeic acid (CAE) in the fruit, was calculated from the equation obtained from the calibration curve of caffeic acid (*y* = 1.7321*x* + 0.0227; *R*2 = 0.9992). Samples were analyzed in triplicate.

### 2.5. Tannin Estimation

The amount of tannin estimation was determined using Pharmacopoeia procedure [20]. The content of tannins was expressed as fresh and dry weight percentage.

### 2.6. Anthocyanins Estimation by means of Colorimetry

Samples of raw material (1.0 g) were extracted with 50 mL HCl (1 mol·dm^3^) and heated in water bath for 1 hour. The obtained extract was hydrolyzed with 20 mL n-butanol, and then two portions of 10 mL n-butanol were added as a solution. Anthocyanin extract was rinsed in 50 mL flask with n-butanol. The absorbance was measured immediately at 533 nm [22]. The percentage of anthocyanins, as delphinidin chloride, was calculated from the expression

(2) $$\begin{array}{c} P=\frac{A\times V\times F}{m}, \end{array}$$

where *P* are total anthocyanins (mg·100 g^−1^); *A* is absorbance at 533 nm; *V* is value of butanol phase (50 mL); *F* is coefficient for delphinidin chloride (2,6); *m* is mass of sample to be examined (mg).

### 2.7. Antioxidant Activity

Antioxidant activity (%) was evaluated on a base of the ability to neutralize the DPPH radicals by means of spectrophotometry according to Chen and Ho [23]: to do this, water extracts were prepared from fruits; extracts were then evaporated till dried and lyophilized. Analyses were performed for 20 *μ*g·mL^−1^ concentration. The absorbance measurements were made at *λ* = 517 nm wavelength using spectrophotometer HITACHI U-2900.

### 2.8. Chemicals

All reagents and solvents were of analytical grade chemicals from Merck (Darmstadt, Germany) or Sigma Chemical Co. (St. Louis, MO, USA) and POCH (Gliwice, Poland).

### 2.9. Statistical Analysis

Achieved results from laboratory experiments were statistically processed by means of variance analysis method and Tukey's confidence intervals at 5% confidence level.

## 3. Results

### 3.1. Principal Physicochemical Parameters of Studied Material

Determinations related to the chemical composition of three wild strawberry cultivars fruits here presented were preceded with the evaluation of general physicochemical parameters, that is, dry matter content in raw fruits and weight loss after drying-moisture content in dried fruits. Data presented in Table 2 indicate that dry matter content in raw fruits ranged from 27.95% to 37.73%. Among compared cultivars, “Baron von Solemacher” fruits were characterized by the highest concentration of the component (36.37%, on average), while “Yellow Wonder” contained the lowest level of the parameter (28.22%). Significant differences between cultivars in subsequent years of study were observed. Considering the water content in dried material, fruits of studied wild strawberry cultivars slightly differed from each other and statistical analysis did not reveal any significant differences. Regardless of the cultivar, moisture content of air-dried fruits oscillated around 11.10%, on average (Table 2).

Among compared cultivars, “Baron von Solemacher” fruits were characterized by the highest concentration of the component (36.37%, on average), while “Yellow Wonder” contained the lowest level of the parameter (28.22%). Significant differences between cultivars in subsequent years of study were observed. Considering the water content in dried material, fruits of studied wild strawberry cultivars slightly differed from each other and statistical analysis did not reveal any significant differences. Regardless of the cultivar, moisture content of air-dried fruits oscillated around 11.10%, on average (Table 2).

### 3.2. Flavonoids Contents

**Figure 1:**
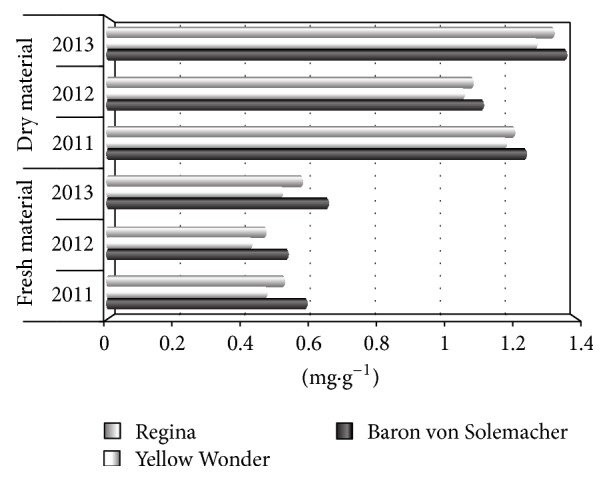
Content of flavonoids (as quercetin) in raw material.

Different levels of flavonoids in raw and dried fruits of three wild strawberry cultivars are presented in Figure 1 and Table 3. The highest concentrations of flavonoids was found in fruits of Baron von Solemacher cv. 0.593 mg·g^−1^ (raw material) and 1.245 mg·g^−1^ (dried material). On the other hand, the lowest quantities of analyzed compounds were recorded in fruits of Yellow Wonder cv. (0.471 and 1.178 mg·g^−1^, resp., for raw and dried material). Significant differences in flavonoids contents over the years of study for both analyzed types of material were observed. The dried fruits of all studied genotypes were characterized by over twice as high flavonoids amounts as compared to raw fruits.

### 3.3. Total Phenolic Acids Contents

**Figure 2:**
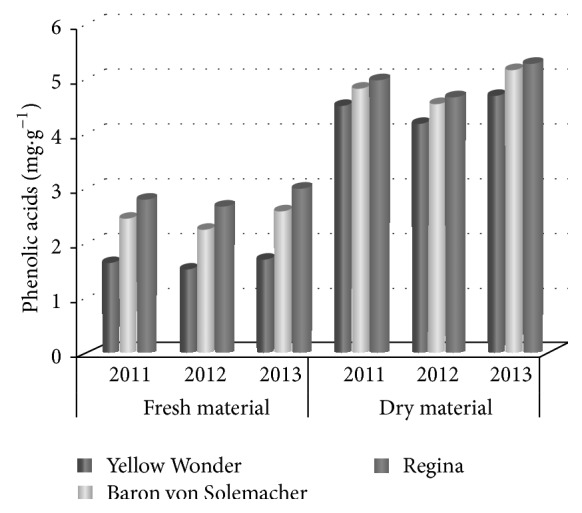
Content of phenolic acids (as caffeic acid) mg·g^−1^ in raw material in successive years of the study.

Analysis of phenolic acids concentration revealed that raw and dried fruits of Regina cv. were the best sources of these compounds 2.840 mg·g^−1^ in raw and 4.987 mg·g^−1^ in dried material (Table 3 and Figure 2).

Slightly less phenolic acids were found in fruits of Baron von Solemacher cv. 2.454 mg·g^−1^ and 4.858 mg·g^−1^ in raw and dried fruits, respectively. Fresh fruits of Yellow Wonder cv. contained almost twice as low phenolic acids as Regina cv. fruits. However, mean values recorded for dried fruits of all analyzed genotypes slightly differed and statistical analysis confirmed that the differences were significant.

### 3.4. Total Tannins Contents

**Figure 3:**
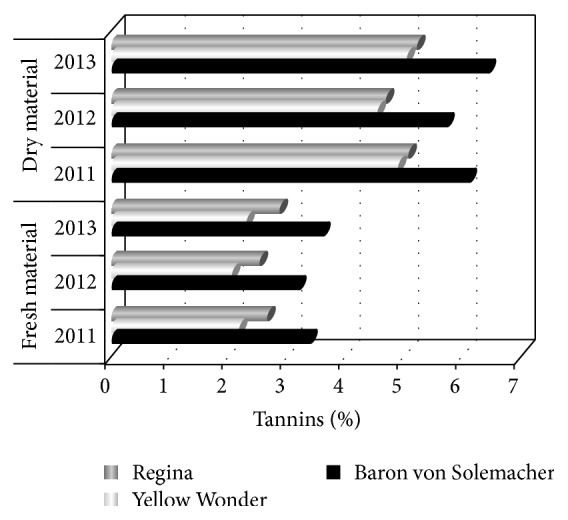
Content of tannins (%) in raw material in successive years of the study.

Changes in tannins contents for all studied wild strawberry fruits were similar as those for flavonoids. Raw fruits contained 2.76% of tannins, on average, while dried ones contained 5.31% (Table 3 and Figure 3). Significant influence of all examined factors on tannins contents in studied materials was found on a base of determinations performed.

### 3.5. Total Anthocyanins Contents

**Figure 4:**
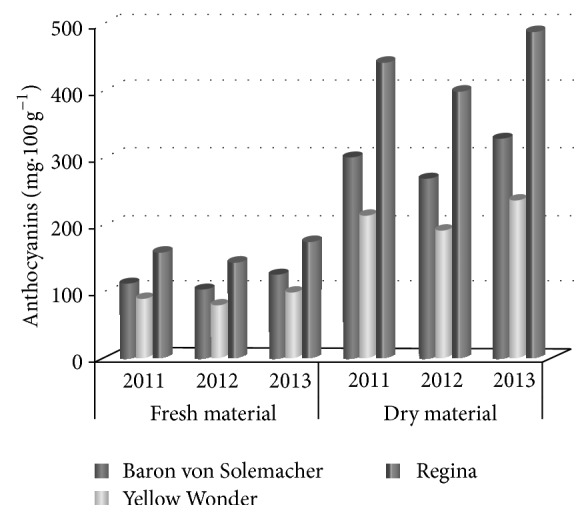
Content of anthocyanins (mg·100 g^−1^) in raw material.

Anthocyanins were another group of substances analyzed in wild strawberry fruits (Table 3 and Figure 4). Fruits of Regina cv. were the best source of anthocyanins as similar as phenolic acids. From 144.12 mg·100 g^−1^ to 177.07 mg·100 g^−1^ were recorded in raw fruits of this cultivar, whereas dried fruits contained from 398.54 mg·100 g^−1^ to 460.40 mg·100 g^−1^. Among compared cultivars, the Yellow Wonder cv. appeared to be the worst since it contained only from 80.97 mg·100 g^−1^ to 99.00 mg·100 g^−1^ (raw material) and from 192.52 mg·100 g^−1^ to 236.90 mg·100 g^−1^ (dried material) of anthocyanins. The lowest levels of analyzed substances for all studied genotypes were recorded in 2012.

### 3.6. Antioxidant Activity

Significant differences in the ability to neutralize the free DPPH (diphenylpicrylhydrazyl) radical by extracts made of examined wild strawberry fruits were recorded (Table 4).

Extracts prepared from dried fruits revealed definitely highest ability (23.93%) as compared to raw material (13.11%). When comparing studied cultivars, the highest neutralizing capacity was shown by extracts made of Regina cv. fruits 14.27% (raw material) and 24.60% (dried fruits). The free radicals were worse reduced by extracts prepared from fruits of Yellow Wonder cv. and Baron von Solemacher cv.

## 4. Discussion

*Fragaria vesca* has traditionally been a popular delicious fruit for its flavor,taste, fresh use, freezing, and processing. Morphological, biometric, and agronomical characteristics have been widely used to describe wild strawberry cultivars. In recent years, cultivated berries have become very attractive for consumers because of potentially beneficial phytochemicals contained in these fruits. The importance of flavonoids and other phenolics has been suggested to play a preventive role in the development of cancer and heart disease [24]. Considerable data suggests that higher content of flavonoids, phenolic acids, tannins, and anthocyanins in berry fruits contributes to their higher antioxidant activity [17, 25]. Following substances should be counted as the most important bioactive components of wild strawberry: phenolic acids (ellagic,* p*-coumaric, gallic acids), flavonoids (flavonols, quercetin, and kaempferol), proanthocyanidins, and anthocyanins (pelargonidin, cyanidin) [26–28]. Due to these compounds, the fruits have anticarcinogenic, antioxidant, anticoagulant, immunomodulating, anti-inflammatory, blood pressure, and cholesterol regulating features [29, 30]. Measuring such parameters is wildly used to evaluate the potential health benefits of breeding material or various agronomic factors [9]. However, little is known on the phenolic profiles and antioxidant potential of wild strawberry in important local cultivars. In this paper, we use the contents of secondary metabolites for cultivar identification. The differences in the concentrations of secondary compounds determine the nutritional importance of the analyzed cultivars. Furthermore, they provide information on the marketing potential of the 3 cultivars and present an important chemical insight into the popular cultivars grown in this region.

Secondary metabolites content in berry fruits varies among species and cultivars, but it can also be affected by growth conditions including environmental factors and cultivation techniques [10, 11, 31–34]. It has been shown that higher growing temperatures (day and night) increase the flavonols and anthocyanins contents in strawberries [26]. Authors of the present study, on a base of performed research, proved differentiated contents of biologically active substances: flavonoids, phenolic acids, tannins, and anthocyanins in raw and dried fruits depending on wild strawberry cultivar. Furthermore, they analyzed the antioxidant ability of extracts made of studied materials by means of neutralizing the free DPPH radical. Mean content of flavonoids and tannins in fresh fruits of “Baron von Solemacher” cv. was the highest and amounted to 1.245 mg·g^−1^ and 6.09%, respectively. Raw fruits of “Regina” cv. were characterized by the highest average concentrations of phenolic acids and anthocyanins: 4.987 mg·g^−1^ and 444.25 mg·100 g^−1^, respectively. According to Antal et al. [35], the quantity of anthocyanins in raw berries ranges from 30 mg·100 g^−1^ (*Fragaria moschata*) to 165 mg·100 g^−1^ (*Vaccinium myrtillus*). Huang et al. [14] found higher contents of anthocyanins in berry fruits, which correspond with values achieved in the present study for raw fruits of examined wild strawberry genotypes. In opinion of Olsson et al. [11], the amount of phenolic substances as well as antioxidant activity was different within* Fragaria *x* ananassa* Duch. genus. Antal et al. [35] evaluated fresh fruits and found that strawberries had total phenolics of 3.680 mg·kg fresh weight. As it follows from performed analyses, mean content of phenolic acids for fresh wild strawberry fruits was at the level of 2.314 mg·g^−1^. The differences in polyphenol content of strawberry fruit from the literature may be due to the different conditions during the growth of plants (climatic conditions, temperature, precipitation, and soil conditions), the length of the growing season, and harvest date. They may also be caused by using various analytical procedures or methods identifying the active ingredients by scientists in various centers. Identification of chromatographic methods usually has a lower content of active substances, because its task is to determine the minimum content and separation and identification of individual compounds in the raw material. Commonly used spectrophotometric procedures are based on similar assumptions, so after taking into account differences methods are comparable to those applied in this paper. During the three years of the research, the highest contents of analyzed biologically active substances were recorded in 2013, while the lowest were recorded in 2012.

In a previous investigation, we found that fresh wild strawberry fruits possess high amounts of bioactive compounds. However, fresh fruits are not available all year round, being harvested in Poland only in June–September. Therefore, it is important to find a proper substitute that could be used when fresh berries are not available. It was decided to prepare dried fruits, to determine the contents of some important bioactive compounds and their antioxidant potential therein, and to compare them with the same parameters in fresh fruits. Dried fruits have a greater nutrient density, greater fiber content, increased shelf life, and significantly greater phenol antioxidant content compared to fresh fruits. The quality of the antioxidants in the processed dried fruit is the same as in the corresponding fresh fruit. Phenols in dried fruit may be important antioxidants [24]. Data presented in Tables 2–4 indicate diverse contents of biologically active compounds in raw and dried materials depending on the cultivar. The highest mean concentrations of analyzed substances in air-dried fruits were similar as for fresh ones. Dried fruits of analyzed genotypes were characterized by over twice as high quantities of biologically active substances and antioxidant activity as fresh fruits. Results from the present study are a confirmation of the results achieved by Vinson et al. [24] related to the secondary metabolites in fresh and dried fruits.

## 5. Conclusion

As a conclusion, our results clearly demonstrate that considerable variation exists in the phenolic compounds among wild strawberry genotypes. The obtained results allowed the identification of cultivars which were characterized by the highest content of investigated biologically active substances and the highest ability to free radicals elimination. Fruits of these cultivars were characterized by the highest prohealth properties. The results from the performed experiments also have practical application during the breeding program preparation in order to obtain new cultivars of this species. Therefore, dried fruits are good source of important bioactive compounds and more dried fruits should be recommended to be added to the diet by dieticians and nutritionists.

## Figures and Tables

**Table 1:** Mean monthly air temperatures, amount, and total hours of precipitation at ES Felin in the years 2011–2013.

Month	2011	2012	2013	Mean for 1951–2000
	Temperature °C			
IV	8.8	8.7	9.3	7.5
V	14.9	15.0	12.8	13.0
VI	18.1	18.1	17.7	16.5
VII	19.1	19.2	18.3	17.9

	Amount of precipitation mm			
IV	17.4	17.4	55.8	40.6
V	80.5	81.5	101.6	58.3
VI	87.8	87.8	25.9	65.8
VII	87.0	87.0	77.1	78.0

	Total hours in sunshine hrs.			
IV	238.8	139.6	291.5	156.6
V	268.6	183.0	274.6	280.9
VI	272.2	316.6	200.3	228.7
VII	236.7	242.3	279.6	158.0

**Table 2:** Dry matter (%) content in raw and dried fruits as well as moisture content in dried fruits in successive years of the study.

Cultivars	Raw material							
	Fresh material				Dry material			
	2011	2012	2013	Mean	2011	2012	2013	Mean
“Baron von Solemacher”	35.20cA	36.19cA	37.73cA	**36.37**	11.36aA	11.33aA	11.24aA	**11.31**
“Yellow Wonder”	28.47aA	27.95aA	28.23aA	**28.22**	11.26aA	11.05aA	11.01aA	**11.11**
“Regina”	31.12bA	30.98bA	31.17bA	**31.09**	11.35aA	10.49aA	10.81aA	**10.88**
Mean	**31.60**	**31.71**	**32.38**	**31.89**	**11.32**	**10.96**	**11.02**	**11.10**

Explanatory notes: different letters a, b, c… and A, B, C… in the same column and line indicate statistically significant differences (*P* < 0.05). In each column and for each cultivar different letters mean significant differences (*P* < 0.05).

**Table 3:** Content of flavonoids TFL (as quercetin) mg·g^−1^, phenolic acids TPC (as caffeic acid) mg·g^−1^, tannins (%), and anthocyanins ACN (as delphinidin mg·100 g^−1^) in raw material.

Cultivars	Raw material							
	Fresh material				Dry material			
	TFL	TPC	TAN	ACN	TFL	TPC	TAN	ACN
“Baron von Solemacher”	0.593c	2.454b	3.40c	114.00a	1.245b	4.858b	6.09b	300.00b
“Yellow Wonder”	0.471a	1.648a	2.19a	90.00a	1.178a	4.483a	4.83a	214.61a
“Regina”	0.524b	2.840b	2.70b	160.50b	1.210ab	4.987b	5.00a	444.25c
Mean	**0.530**	**2.314**	**2.76**	**121.50**	**1.211**	**4.776**	**5.31**	**319.62**

Explanatory notes: see Table 2.

**Table 4:** Antioxidant activity (%) expressed as the ability to neutralize the DPPH radical in water extracts made of studied materials in successive years of the study.

Cultivars	Raw material							
	Fresh material				Dry material			
	2011	2012	2013	Mean	2011	2012	2013	Mean
“Baron von Solemacher”	12.65a	11.34a	14.02b	**12.67**	23.84a	21.51b	26.05b	**23.80**
“Yellow Wonder”	12.42a	11.16a	13.63a	**12.40**	23.44a	21.05a	25.70a	**23.40**
“Regina”	14.30b	13.21b	15.30c	**14.27**	24.66b	22.15c	26.98c	**24.60**
Mean	**13.12**	**11.90**	**14.31**	**13.11**	**23.98**	**21.57**	**26.24**	**23.93**

Explanatory notes: see Table 2.
